# Fetal Fractures in an Infant with Maternal Ehlers-Danlos Syndrome, CCDC134 Pathogenic Mutation and a Negative Genetic Test for Osteogenesis Imperfecta

**DOI:** 10.3390/children8060512

**Published:** 2021-06-17

**Authors:** Michael F. Holick, Arash Shirvani, Nipith Charoenngam

**Affiliations:** 1 Section Endocrinology, Diabetes, Nutrition and Weight Management, Department of Medicine, Boston University School of Medicine, Boston, MA 02118, USA; hn@bu.edu (A.S.); ncharoen@bu.edu (N.C.); 2 Department of Medicine, Faculty of Medicine Siriraj Hospital, Mahidol University, Bangkok 10700, Thailand

**Keywords:** osteogenesis imperfecta, multiple fractures, intrauterine fracture, infantile fracture, posterior rib fractures, Ehlers–Danlos syndrome, genetic mutation, nonaccidental trauma, child abuse

## Abstract

Intrauterine fractures are a rare clinical finding caused by abnormal early-life osteogenesis. In this case report, we reported a male infant with twenty-three intrauterine/fetal fractures resembling osteogenesis imperfecta and tested negative for *COL1A1* and *COL1A2* mutations. The infant’s mother had Ehlers–Danlos syndrome, hypermobility type. Whole-genome sequencing revealed that there were no pathologic mutations previously documented to be associated with intrauterine fracture. Genetic mutations reported to be associated with fragility fractures were identified. These include the pathogenic homozygous mutation in the *CCDC134* gene. Other genetic variants that might be responsible for variable expressivity of the skeletal manifestation include the homozygous variants of the genes *CCDC134*, *COL15A1* and *ZFPM1*, and the heterozygous variants of the genes *MYH3, BCHE, AUTS2*. This is the first reported case of in utero fractures, that was confirmed by X-ray after birth, in an infant who had no genetic evidence for osteogenesis imperfecta, had a homozygous pathogenic mutation of an osteogenesis gene and whose mother had Ehlers-Danlos syndrome hypermobility type. Therefore, we have identified a new genetic cause for in utero fractures. If after birth, this infant were found to have these fractures in various stages of healing with a negative genetic test for osteogenesis imperfecta he would have been misdiagnosed as due to nonaccidental trauma.

## 1. Introduction

Intrauterine fractures are a rare finding in routine prenatal imaging studies. This condition can be secondary to maternal trauma, genetic disorders of the skeleton, as well as other predisposing maternal metabolic and vascular disorders [1]. Genetic disorders that have previously been reported to cause intrauterine fractures include osteogenesis imperfecta (OI), osteopetrosis, hypophosphatasia and Ehlers–Danlos syndrome (EDS) type VII with a genetic mutation of type I collagen [1]. Other acquired factors that may increase the risk of intrauterine skeletal fragility include vascular compromise of the fetal skeleton and maternal metabolic abnormalities [1].

OI is the most common hereditary bone fragility disorder associated with abnormalities in type I collagen causing a variety of clinical manifestations [2]. At least 90% of OI patients have autosomal dominant mutations in *COL1A1* or *COL1A2* genes, while the minority of the patients had other mutations that affect structural integrity of the skeleton [2,3]. Interestingly, it has been shown in some populations that a significant number of patients with clinical features of OI tested negative for known causative genetic mutations for OI [3,4]. These observations suggest that when an infant presents with a history of a fracture or fractures with a negative genetic test for OI, there are likely causes besides nonaccidental trauma, including other causative genetic disorders of the structural components of the skeleton, resulting in bone fragility. 

Like OI, Ehlers–Danlos Syndrome (EDS) is a genetic disorder of the collagen–elastin matrix. However, unlike OI, most forms of EDS, including EDS, hypermobility type (hEDS), are not associated with mutations of either *COL1A1* or *COL1A2* genes, and the causative genetic mutation of hEDS is still unknown [5,6]. Many of the physical manifestations of OI are very similar to those observed in patients with EDS, including capillary fragility, joint hypermobility and bone fragility in infants and adults [5,7]. Other clinical manifestations of hEDS include chronic pain, mast cell hypersensitivity, gastroparesis, chronic fatigue, dysautonomia, and anxiety among other associated symptoms. The management of hEDS includes treatment of acute manifestations such as joint dislocation, attenuation of chronic symptoms, and prevention of acute and chronic complications [6]

We report a male infant who had multiple fractures in utero consistent with OI features. Genetic testing for OI was negative. The patient’s mother had been previously diagnosed with hEDS. We identified a homozygous initiator codon loss-of-function mutation in the *CCDC134* gene along with other possible predisposing genetic variants, including the homozygous variants of the genes *CCDC134*, *COL15A1* and *ZPFM*, and the heterozygous variants of the genes *MYH3, BCHE, AUTS2* and *ZFPM1*. We discussed how the combination of these variants may cause the complex phenotype of OI and EDS features.

### Case Report

**Figure 1:**
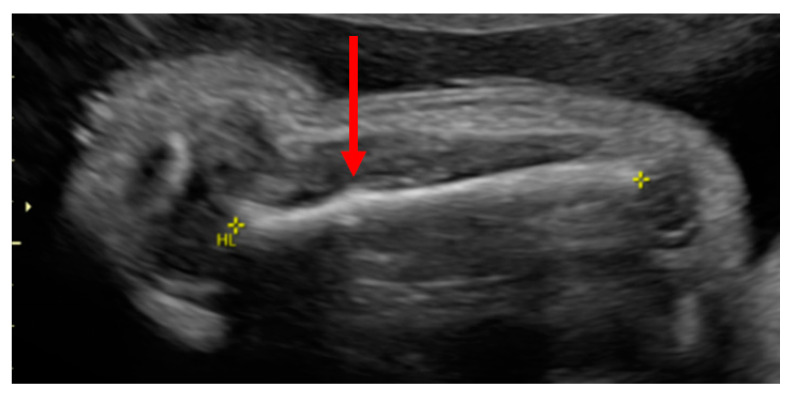
Prenatal ultrasound at 32w1d of gestation. The fetal survey showed a transverse linear fracture line through the proximal right humeral diaphysis with some associated bowing and callus formation. Holick MF, copyright 2021.

**Figure 2:**
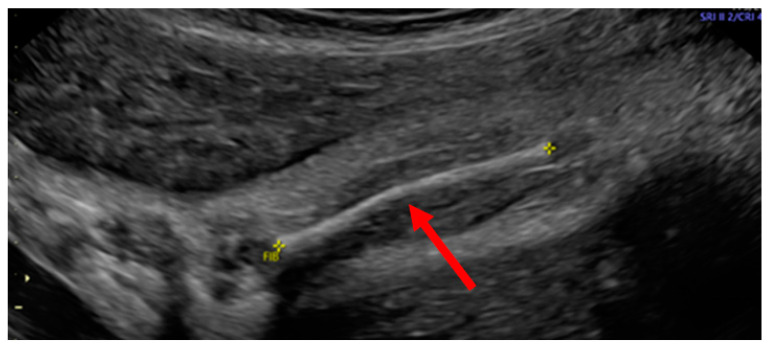
There was mild anterior bowing of the left fibula and, to a lesser extent, tibia. Holick MF, copyright 2021.

**Figure 3:**
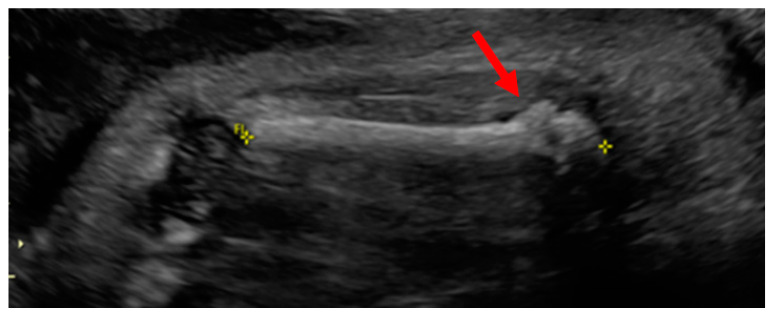
There was angulation at the femoral metadiaphysis bilaterally with callus formation at the metadiaphysis of the left femur (arrow, Figure 3). Holick MF, copyright 2021.

The patient, at 32w1d of gestation, was found to have intrauterine growth retardation (IUGR), a decreased thoracic size, short limbs and multiple fractures by high-resolution ultrasonography (Figure 1, Figure 2 and Figure 3). The ultrasound performed by a licensed technician and interpreted by a board-certified radiologist showed a placenta posterior that was normal in appearance. A subsequent fetal MRI revealed a deformity of the thoracic cage and micromelia with suspected bilateral fractures of the proximal femurs and the right upper extremities. These findings suggested a variant of OI or a syndrome resembling OI. 

**Figure 4:**
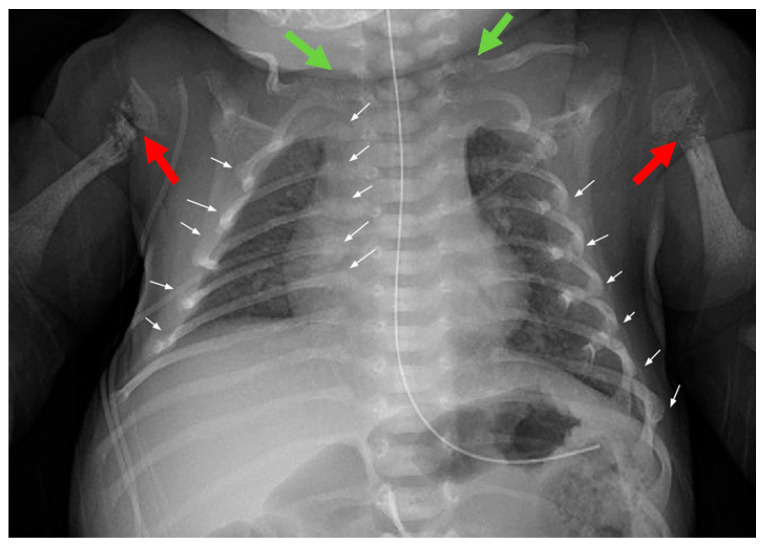
The chest X-ray at 7 days of age showed bilateral fractures of the clavicular heads (green arrows) and likely multiple posterior rib fractures, including the right 2nd–5th ribs, the right lateral 2nd–7th ribs, and the left 3rd–9th ribs (white arrows). Multiple fractures involved the bilateral proximal humeral diaphyses with poorly formed callus at the fracture margins and impaction of the fractures (red arrows). Holick MF, copyright 2021.

**Figure 5:**
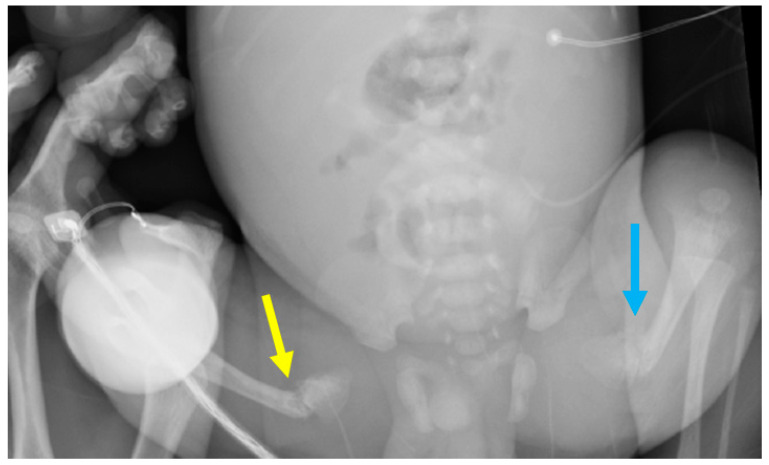
The pediatric long-bone survey taken on the day of birth indicated a displaced, healing fracture of the left proximal femoral diaphysis, with a 7 mm posterior displacement of the distal fragment (blue arrow). There was a partially imaged healing fracture of the right proximal femur (yellow arrow). Holick MF, copyright 2021.

**Figure 6:**
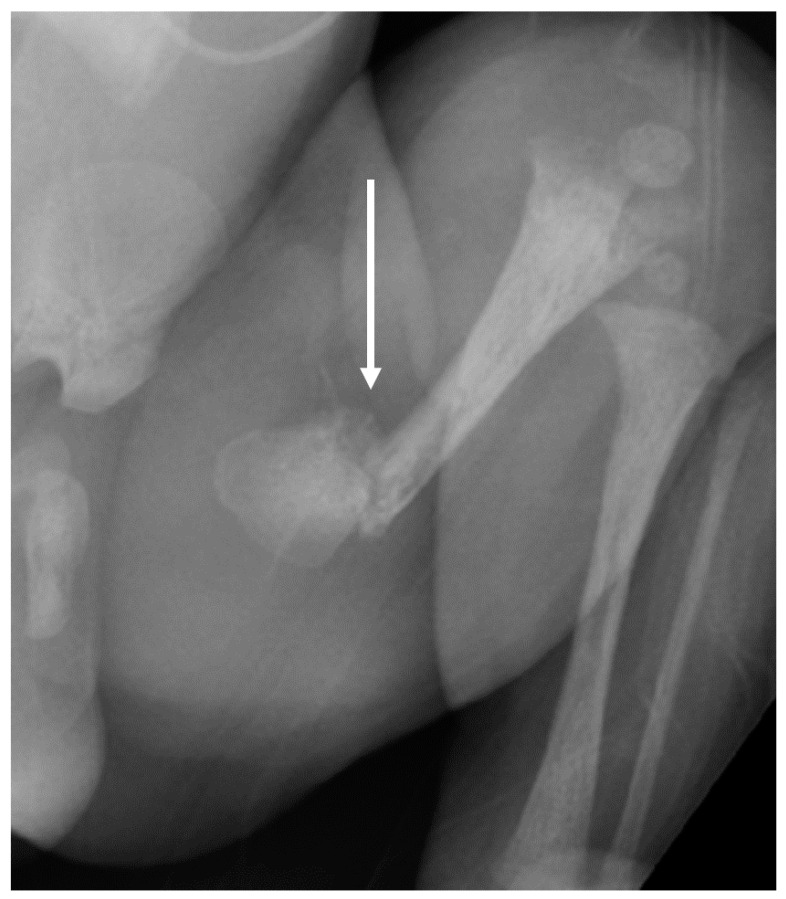
On the day of birth, there was surrounding callus formation and sclerosis surrounding the margins of the fracture of the left proximal femur (white arrow). There was a cortical irregularity in the distal metaphysis of the left femur, with faint lucency and no displacement of fragments. Holick MF, copyright 2021.

The male infant was born at 40w1d gestational age via C-section due to prolonged labor with no significant complications at Boston Medical Center. Placental examination was unremarkable. The patient’s birth weight of 3030 g was at the 19th percentile and his length was 45 cm (below the 3rd percentile). The occipitofrontal head circumference was 36 cm (77th percentile). After birth, the radiologic findings of fractures were consistent with the ultrasonography during pregnancy. These included multiple rib fractures and bilateral fractures of the proximal humeral and femoral diaphysis (Figure 4, Figure 5 and Figure 6). He was then transferred to Boston Children’s Hospital for bisphosphonate therapy, further management, and genetic testing. A subsequent genetic test for OI was negative for pathogenic variants.

## 2. Materials and Methods

The mother, previously diagnosed with hEDS, and her family participated in the Boston University Medical Campus’s Institutional Review Board-approved Ehlers–Danlos Clinical Research Program registered on clinicaltrials.gov (NCT03093493). After signing the IRB-approved consent forms, each parent was given a questionnaire to report medical history and symptoms related to EDS and related hereditary connective tissue disorders for themselves and their infant. A physical examination was performed to evaluate signs of EDS and related connective tissue disorders [8]. DNA was extracted from buccal swabs for whole-genome sequencing. The results were filtered based on prespecified criteria for frequency, functionality and pathogenicity. Candidate genetic variants included known variants in the coding area of the genes that are related to the collagen matrix or bone metabolism, as well as variants that are known to be associated with EDS, OI or other hereditary connective tissue disorders. 

### Whole-Genome Sequencing

The genetic variations in DNA samples of the infant and his parents were evaluated by whole-genome sequencing (WGS) at the Molecular Biology Core at the Dana–Farber Cancer Institute. After initial sample QC, an automated PCR-free library preparation was performed using the Swift 2S protocol, and a 60X whole-genome sequencing of 100 bp paired-end reads was carried out on a HiSeq 2000 (Illumina, Inc., San Diego, CA, USA). Fast QC was used to evaluate the quality of the reads. BWA-MEM was used for mapping the reads to the GRCh38 reference of human sequence.

The data was uploaded to Illumina BaseSpace for final analysis. We completed running Illumina’s Isaac-based whole-genome sequencing pipeline in BaseSpace with our WGS samples and generated VCFs for small variants. The small variant VCFs were then imported into the Variant Interpreter, which performed some basic annotation and filtered for PASS variants. The final results of the WGS analysis were verified by Sanger sequencing. Sequencing primers are available upon request.

## 3. Results

### 3.1. Phenotypes of the Patient and Parents

After birth, the skeletal survey confirmed multiple fractures observed in utero (Figure 2). The mother was a 34-year-old, G2A1 healthy woman who immigrated from Morocco. She, her son and husband were seen 2 months after her pregnancy. She had been previously diagnosed with hEDS and had a history of easy bruisability, flushing without provocation, joint hypermobility, recurrent joint subluxations/dislocations, gastroparesis and chronic musculoskeletal pain. In addition, she had a remote history of a nonunion fracture of her humerus which was treated with 4000 IUs per day of vitamin D_3_ and teriparatide. She did not have any history of significant trauma during her pregnancy. She took 4000 IUs per day of vitamin D_3_ during her pregnancy. Her 25-hydroxyvitamin D level was 49 ng/mL, consistent with vitamin D sufficiency. Her mineral status and bone turnover markers were normal in her second trimester of pregnancy (albumin-corrected calcium 9.5 (normal range 8.0–10.5 mg/dL), phosphate 3.0 (normal range 2.7–4.5 mg/dL), intact parathyroid hormone 35 (normal range 11–90 pg/mL), urine N-telopeptide 29 (normal range 4–64 nmol BCE/mmol creatinine), osteocalcin 11 (normal range 8–32 ng/mL)). On physical exam, she presented with grey–blue sclera, a grade I/VI systolic heart murmur with click, doughy-textured, velvety skin with hyperextensibility, increased translucency and abnormal scarring. She had significant joint hypermobility of her fifth digits and elbows and could touch the floor with the palms of her hands, giving her a Beighton score of 5/9 [8]. Her physical findings fulfilled the major criteria (i.e., skin involvement and generalized joint hypermobility with a Beighton score of 5/9 or greater) and two of the minor criteria (i.e., recurrent joint dislocations and chronic joint/limb pain). The medical history and physical examination of the father, who is Moroccan, did not reveal any evidence for EDS, Marfan’s syndrome, OI or other genetic causes for metabolic bone disease. There was no history of consanguinity in the family.

On examination at six months of age, the infant had intense blue sclera and very transparent and mottled skin. Physical examination of extremities confirmed the proximal humeral and femoral bowing, flexed wrist, and large-appearing hands seen on ultrasound. Significant joint hypermobility of the wrists, elbows and hip was observed. 

### 3.2. Whole-Genome Sequencing Results

Total reads were between 850 million and 1 billion base pairs, and the percentage of aligned reads/bases were 84–86%. The percentage of Q30 bases was 91–94%, which indicates good quality of the whole-genome sequencing. 

**Figure 7:**
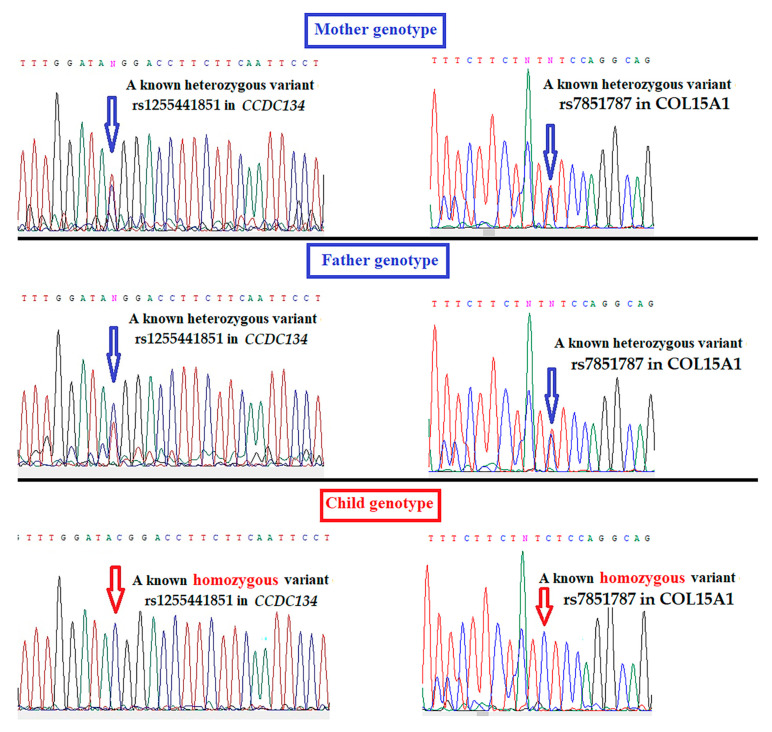
Sequencing chromatograms demonstrating the genetic variants of the *CCDC134* and *COL15A1* genes found in the family.

A total of 5,477,300 genetic variants were determined in the infant. Filtering for small variants, coding consequences and prediction of pathogenic variants, likely pathogenic variants and variants of uncertain significance using the Variant Interpreter (Strelka 2.9.2, Illumina, Inc. San Diego County, CA, USA) revealed 28 candidate variants. None of them were associated with pathologic variants that have previously been documented in EDS, OI, other genetic disorders causing intrauterine or infantile skeletal fragility, undermineralization or micromelia, including achondrogenesis, hypophosphatasia or vitamin D-resistant rickets. Each of the 28 variants passed QC filters and was then checked for quality manually. Among them, 11 variants were likely benign or tolerated results in Sift or PolyPhen prediction and were excluded from the analysis. This left 17 filtered variants (Table 1 and Table 2). Among these 17 identified variants, variants of the *CCDC134*, *COL15A1, MYH3, BCHE, AUTS2* and *ZFPM1* genes were found to be significantly involved in osteogenesis based on extensive literature review of the functionality of each identified variant and/or gene. Sanger sequencing confirmed the whole-genome sequencing results (Figure 7). 

## 4. Discussion

This is the first case report of an infant with multiple intrauterine fractures of long bones and anterior and posterior ribs consistent with a phenotype of OI, who tested negative for genetic mutations of type I collagen (i.e., *COL1A1* and *COL1A2* genes). This was confirmed by our whole-genome sequencing evaluation. Our clinical evaluation of the mother was consistent with her diagnosis of hEDS. Although it can be difficult to determine if an infant has EDS hypermobility type since there is no genetic test for this condition, the infant had many physical characteristics and medical history conditions associated with EDS hypermobility type. These included blue sclerae, mast cell hypersensitivity, gastroparesis symptoms, excessive joint hypermobility, and greater skin translucency and elasticity than would be expected for a six-month-old child. Whole-genome sequencing of the infant and his parents’ DNA did not reveal any pathologic mutations known to cause OI or classical EDS. However, whole-genome sequencing of the infant revealed potentially pathogenic variants associated with osteogenesis and bone development, including the homozygous variants of the genes *CCDC134*, *COL15A1* and *ZFPM1,* and the heterozygous variants of the genes *MYH3, BCHE* and *AUTS2*. Based on these findings, it can be concluded that the multiple intrauterine fractures in this infant may be caused by the combination of these genetic variants. Of note, although there is no history of consanguinity in the family, this possibility cannot be excluded based on the observed identical rare variants.

The underlying pathophysiology of the multiple intrauterine fractures observed in this infant is thought to be primarily mediated by the dysregulated ERK-MAPK pathway in the osteoprogenitors, which has been shown to be essential for skeletal development and homeostasis [9]. The *CCDC134* gene encodes the coiled-coil domain containing 134 (CDCC134) secretory protein that inhibits the intracellular ERK-MAPK pathway by inhibiting transcriptional activity of ELK1 and phosphorylation of ERK and JNK/SAPK [10]. Dubail et al. [3] reported that the homozygous loss-of-function mutation at the initiator codon in the *CDCC134* gene caused bone fragility in three patients who presented with clinical features of OI which did not respond to bisphosphonate therapy. Subsequent functional studies confirmed that this loss-of-function genetic mutation of *CDCC134* leads to the absence of the CDCC134 protein, which induced the phosphorylation of ERK and inhibited the expression of *OPN* (osteopontin) and *COL1A1*, thereby leading to reduced mineralization in the osteoblasts of the patients [3]. It should be noted that one of the patients in the Dubail paper was found to have fractures at birth. It is unclear if these were new fractures from the birth process or healing fractures, in which case the phenotype would be similar to our patient, who had as many as 23 in utero/fetal fractures.

While the mutation of *CDCC134* is likely the most important causative factor for in utero skeletal fragility in our patient, it should be noted that this genetic mutation has been previously reported to have highly variable phenotypic expressivity, including clinical features of bone fragility/OI in childhood and adulthood [3]. It is therefore probable that other genetic variants identified in our patient may have contributed to the development of a more severe form of skeletal fragility that led to intrauterine fractures. These include the homozygous variants of *COL15A1* and *ZFPM1,* and the heterozygous variants of *MYH3, BCHE* and *AUTS2*. Although the exact mechanisms by which the genetic variations in these genes affect early-life skeletal development are still unclear, there is evidence that these genes are involved in the osteogenic process and maintenance of the healthy mineralized skeleton. 

*COL15A1* encodes the alpha chain of type XV collagen that is recently known as novel bone extracellular matrix protein [11]. This protein plays an essential role in the early stage of the osteogenic process and has been implicated in bone mineralization by influencing the deposition of minerals into the matrix [11]. *ZFPM1* encodes the zinc finger protein, FOG family member 1. This protein interacts with the transcription factor GATA2 in the osteogenic lineage and was shown to be essential for trabecularization and the mechanical strength of the bone [12]. *MYH3* encodes the protein myosin heavy chain 3, a major contractile protein in the skeletal muscle [13]. Genetic mutations in this gene are associated with congenital arthrogryposis syndromes and spondylocarpotarsal synostosis syndrome, a rare group of skeletal dysplasias [14,15]. The relationship between *MYH3* and osteogenesis is thought to be related to its function in regulating transforming growth factor-β activity in the sclerotome [15]. *BCHE* encodes the enzyme butyrylcholinesterase which degrades acetylcholine in addition to acetylcholinesterase. This enzyme is expressed by the osteoblast-like cells and is involved in regulating the number of osteoclasts and bone microarchitecture [16]. Finally, *AUTS2* encodes the autism susceptibility candidate 2 protein that is involved in neural migration and neurogenesis. It has been reported that mutations in this gene are associated with neurological and skeletal abnormalities [17], suggesting the possible link of this gene to skeletal development.

It is of particular interest that the mother was clinically diagnosed with hEDS and the infant therefore had a 50% chance of acquiring it from her. He demonstrated physical findings consistent with hEDS, including joint hypermobility, intense blue sclera, and increased translucency and elasticity of the skin. It is still to be determined whether the infant will continue to demonstrate signs and symptoms of hEDS later in his life, as clinical evaluation of joint hypermobility, physical exam findings and medical history in infants are questioned as to their reliability [18]. 

EDS and OI are connective tissue disorders involving the collagen–elastin matrix that have overlapping clinical features, including bone fragility [19]. Among the 13 subtypes of EDS with different phenotypes, the most common subtype is hEDS, which is associated with joint hypermobility, increased elasticity of the skin, and fragility of the capillaries and skeleton [18]. While there are genetic tests for some subtypes of EDS, no genetic test has been developed for diagnosing hEDS [2,5,18]. EDS has, however, been shown to be associated with an increased risk of fractures in adults, especially of the vertebrae, independent of bone mineral density [20]. In addition, there has been a report of 67 infants with fragility fractures and a concurrent family history of hEDS [21]. Since genetic mutations directly responsible for skeletal fragility in EDS have yet to be identified, the association of EDS with fragility fractures in infants needs more investigation [22,23]. At the same time, it is recognized that EDS and OI present with similar clinical features, including joint hypermobility and vascular fragility [2,21,24,25]. This concept of overlapping clinical features of EDS and OI is supported by the case reports of the coexistence of OI and EDS and is strengthened by our report [21,26]. 

Based on this notion, there may be common genetic mutations that explain bone fragility and joint hypermobility in EDS and possibly OI. Our case report suggests that the genetic mutation in *CCDC134* is one of the possibilities. Although this hypothesis is opposed by the fact that the father had no joint hypermobility, it is not uncommon for a genetic disease to have variable clinical manifestations. Further studies are required to investigate whether the heterozygous mutation of CCDC134 is associated with joint hypermobility. 

This case report has major implications for the approach to the diagnosis of nonaccidental trauma (child abuse) in children with multiple fractures. The findings of multiple fractures with various stages of healing with a negative genetic test for OI, Menkes disease and glutaric acidemia type 1 have been accepted by some geneticists and child abuse experts to be sufficient to support a diagnosis of nonaccidental trauma [27]. The findings in this case imply that the genetic variants involved in skeletal development and fragility are not limited to the current panel of genetic tests and thus raise a question on the validity of the current recommendations. This is consistent with previous reports showing that a large number of patients with clinical features of OI tested negative for known causative genetic mutations for OI [3,4,28,29]. Our report documents impressive in utero fractures of both arms and both legs with follow-up X-rays after birth documenting these fractures as well as anterior and posterior rib fractures in various stages of healing. If this mother had brought in her son for medical care later in his infancy without prior diagnosis of in utero fractures, these X-ray findings would almost certainly have resulted in the diagnosis of nonaccidental trauma since the infant tested negative for OI and other metabolic causes for infantile skeletal fragility, including Vitamin D deficiency. This would likely have resulted in the removal of the infant (and any siblings) from the home and accusations of felony child abuse against the parents. 

This scenario was documented to have occurred in three nonaccidental trauma index cases where at least one parent was documented to have EDS and the infant had medical and physical evidence for the same bone fragility disorder [21]. Similar to our infant who had multiple fractures identified in utero and in infancy and who was vitamin D sufficient, a 2-month-old infant presented with 10 fractures, including a femoral fracture and anterior rib fractures, which were diagnosed as caused by nonaccidental trauma due to the fact that the infant tested negative for OI and the fractures were in various stages of healing. The infant’s mother had EDS and the infant manifested clinical signs for the same genetic disorder. No other metabolic abnormalities were observed in the infant and the infant was found to be vitamin D sufficient. The parents were charged with felony child abuse and the infant removed from their care. After further consideration, the child was returned to the parents [21].

These cases raise serious concern about the present criteria that are used for the diagnosis of nonaccidental trauma in infants who present with multiple fractures. It should be acknowledged that the current diagnostic panel of bone fragility disorder represents only “known” genetic variants, not all possible genetic variants. This notion is supported by the observation by Dubail et al. [3] that 25 of the 350 patients with clinical features of bone fragility consistent with OI remained without molecular diagnosis as they tested negative for OI and related bone fragility disorders. One should consider adding CDCC134 to the genetic panel for bone fragility disorders. This also supports the urgent need for further investigations to identify additional causative genetic variants for skeletal fragility, including yet to be identified genes associated with a well-recognized bone fragility disorder associated with a genetic defect of the collagen–elastin matrix: EDS. 

There are certain limitations of this case report that should be acknowledged. First, a case report generally provides a relatively low level of evidence compared with other study designs. Further studies with a more robust study design are warranted to confirm our observation. Second, we were unable to acquire bone tissue and measure the expression of the *CCDC134* gene in our reported case. However, our results revealing the identical genetic mutation of *CDCC134* in our infant with intrauterine fractures has verified the functional study by Dubail et al. [3] that a dysregulated ERK-MAPK pathway due to CDCC134 deficiency is involved in the pathogenesis of skeletal fragility in these patients. Finally, it should be noted that the *CCDC134* gene is also important in regulating collagen synthesis and promoting proliferation and activation of cytotoxic T lymphocytes [28]. We did not observe any other extra-skeletal abnormality in this infant and the information on T cell function is unavailable. A long-term follow-up of immune function is therefore warranted.

## 5. Conclusions

We report for the first time an infant with multiple intrauterine fractures consistent with a phenotype of OI whose mother has documented hEDS. We have identified potential and possible causative genetic variants in *CCDC134* along with other genetic variants that might be responsible for variable expressivity of the skeletal manifestation, including the homozygous variants of the genes CCDC134, COL15A1 and ZFPM1, and the heterozygous variants of the genes MYH3, BCHE, AUTS2. The whole-genome sequencing also revealed genetic variants that might be causative for hEDS in the mother. 

This is the second report of severe skeletal fragility associated with multiple fractures in infants who tested negative for OI but had the phenotype for OI as well as a homozygous pathologic mutation of the *CCDC134* gene. This severe phenotype could be mistaken for child abuse since these infants tested negative for OI. It is also possible that an infant or child with a pathologic but less deleterious CCDC134 mutation might be without fractures at birth but would present with unexplained fractures later in early infancy/childhood and then be diagnosed as a victim of child abuse. At this time, consideration should be given to adding CCDC134 to the current genetic panel. These findings should also give pause to the diagnosis of nonaccidental trauma in infants or children with fractures characteristic of OI but with negative OI testing. In such cases, the differential diagnosis should be expanded to include other genetic or acquired disorders, including those associated with EDS, vitamin D deficiency and mutations of osteogenesis controlling genes. In these settings, fractures can occur without the application of force. In young infants, one must also consider that the fractures may have occurred or originated in utero. 

## Figures and Tables

**Table 1:** Phenotype and genotypes of the family.

	Mother	Father	Infant
Phenotype	hEDS and nonunion fracture	Normal	Intrauterine Fractures
Gene	Genotypes	Genotypes	Genotypes
*CCDC134*	N/V	N/V	V/V
*COL15A1*	N/V	N/V	V/V
*ZFPM1*	N/V	N/V	V/V
*SNTB1*	N/V	N/V	V/V
*F13B*	N/N	N/V	N/V
*TTN*	N/N	N/V	N/V
*ACAD9*	N/V	N/N	N/V
*BCHE*	N/N	N/V	N/V
*NBEAL2*	N/N	N/V	N/V
*AUTS2*	N/V	N/N	N/V
*ASPH*	N/V	N/N	N/V
*NCAPD3*	N/N	N/V	N/V
*CABP4*	N/N	N/V	N/V
*MYH3*	N/V	N/N	N/V
*KIR2DL3*	N/N	N/V	N/V
*CRYBB3*	N/V	N/N	N/V
*ZNF75D*	N/V	N/N	N/V

Abbreviation: hEDS—Ehlers–Danlos syndrome, hypermobility; N—Normal allele; V—Variant allele.

**Table 2:** The filtered variants in the infant with multiple intrauterine fractures.

Zygosity of Variant in the Infant	Gene	SNP	HGVSC	HGVSP	Allele Frequency	Allele	Clinical Significance	Consequence	Suggested Gene Function	SIFT	PolyPhen
Homozygous variants	CCDC134 *^a^	rs1255441851	c.2T>C	p.Met1Thr	0.000004	T > C	Not reported	Initiator codon loss-of-function	Regulating ERK-MAPK pathwayOsteogenesis Imperfecta phenotype	Deleterious	Probably damaging
	COL15A1 *	rs7851787	c.1762-6T>C	-	0.15361	T > C	Not reported	Splice region variant	Bone extracellular matrix protein	-	-
	ZFPM1 *	rs759189176	c.1335_1338del	p.Leu446fs	0.0003	delTCTG	Not reported	Frameshift variant	Regulating osteogenic lineage by interaction with GATA2		
	*SNTB1*	rs547154887	c.12_14GGC	p.Ala8_Ala10del	0.02	delGCC	Not reported	Inframe deletion	Unknown	-	-
Heterozygous variants	*F13B*	rs17514281	c.1025T>C	p.Ile342Thr	0.007	A > G	Conflicting	Missense variant	Unknown	Deleterious	Probably damaging
	*TTN*	rs397517630	c.57586C>G	p.Leu19196Val	0.00017	G > C	Conflicting	Missense variant	Unknown	-	Probably damaging
	*ACAD9*	rs863224844	c.359del	p.Phe120fs	0.0001	delT	Likely pathogenic	Frameshift variant	Unknown	-	-
	BCHE *	rs1799807	c.293A>G	p.Asp98Gly	0.012	T > C	Likely pathogenic	Missense variant	Regulating the number of osteoclasts and bone microarchitecture	Deleterious	Probably damaging
	*NBEAL2*	rs201373710	c.1948G>A	p.Gly650Arg	0.0016	G > A	Conflicting	Missense variant	Bone marrow	Deleterious	Probably damaging
	AUTS2 *	rs767529359	c.1295C>T	p.Pro432Leu	0.0002	C > A	Conflicting	Missense variant	Skeletal anomalies	Deleterious	Probably damaging
	*ASPH*	rs80163539	c.518del	p.Asp173fs	0.001	delT	Conflicting	Frameshift variant	Osteogenic differentiationunknown	-	-
	*NCAPD3*	rs151013524	c.1981G>T	p.Asp661Tyr	0.0019	C > A	Uncertain significance	Missense variant	Abnormal development of lower spine	Deleterious	Probably damaging
	*CABP4*	rs146764702	c.547G>C	p.Gly183Arg	0.0005	G > C	Uncertain significance	Missense variant	Unknown	Deleterious	Probably damaging
	MYH3 *	rs557849165	c.-9+1G>A	-	0.00274	C > T	Pathogenic	Splice donor variant	Skeletal dysplasia	-	-
	*KIR2DL3*	rs193921051	c.71-4C>T	-	0.08	C > T	Uncertain significance	Splice region variant	Bone marrow	-	-
	*CRYBB3*	rs147937174	c.584G>A	p.Arg195His	0.0001	G > A	Conflicting *	Missense variant	Unknown	Deleterious	Probably damaging
Hemizygous variants	*ZNF75D*	rs150700463	c.934C>T	p.Gln312Ter	0.00134	G > A	Not reported	Stop gained	Unknown	-	-

*—Denotes genes involved in osteogenesis and bone development; ^a^ —The *CCDC134* mutation is an initiator codon variant. Using the Mutation Taster, it is indicated that the possible effect of this mutation is disease-causing by activation of the potential downstream translation initiation site with the same reading frame, resulting in the missing 17 amino acids at the beginning of the sequence. Reproduced with permission from Holick MF, copyright 2021.

## Data Availability

Data will be available beginning 9 months and ending 36 months after article publication upon reasonable request to mfholick@bu.edu.
